# Access to care and frequency of detransition among a cohort discharged by a UK national adult gender identity clinic: retrospective case-note review

**DOI:** 10.1192/bjo.2021.1022

**Published:** 2021-10-01

**Authors:** R. Hall, L. Mitchell, J. Sachdeva

**Affiliations:** Devon Partnership Trust, UK; Plymouth Hospitals Trust, UK; Devon Partnership Trust, UK

**Keywords:** Transgender, transsexual, gender identity, gender dysphoria, detransition

## Abstract

**Background:**

UK adult gender identity clinics (GICs) are implementing a new streamlined service model. However, there is minimal evidence from these services underpinning this. It is also unknown how many service users subsequently ‘detransition’.

**Aims:**

To describe service users’ access to care and patterns of service use, specifically, interventions accessed, reasons for discharge and re-referrals; to identify factors associated with access; and to quantify ‘detransitioning’.

**Method:**

A retrospective case-note review was performed as a service evaluation for 175 service users consecutively discharged by a tertiary National Health Service adult GIC between 1 September 2017 and 31 August 2018. Descriptive statistics were used for rates of accessing interventions sought, reasons for discharge, re-referral and frequency of detransitioning. Using multivariate analysis, we sought associations between several variables and ‘accessing care’ or ‘other outcome’.

**Results:**

The treatment pathway was completed by 56.1%. All interventions initially sought were accessed by 58%; 94% accessed hormones but only 47.7% accessed gender reassignment surgery; 21.7% disengaged; and 19.4% were re-referred. Multivariate analysis identified coexisting neurodevelopmental disorders (odds ratio [OR] = 5.7, 95% CI = 1.7–19), previous adverse childhood experiences (ACEs) per reported ACE (OR = 1.5, 95% CI = 1.1–1.9), substance misuse during treatment (OR = 4.3, 95% CI = 1.1–17.6) and mental health concerns during treatment (OR = 2.2, 95% CI 1.1–4.4) as independently associated with accessing care. Twelve people (6.9%) met our case definition of detransitioning.

**Conclusions:**

Service users may have unmet needs. Neurodevelopmental disorders or ACEs suggest complexity requiring consideration during the assessment process. Managing mental ill health and substance misuse during treatment needs optimising. Detransitioning might be more frequent than previously reported.

‘Trans’ is an umbrella term describing those whose gender identity differs from their natal sex. Some people with trans gender identities experience clinically significant distress and secondary impairment in function and are diagnosed with ‘gender dysphoria’; they may go on to seek medical and surgical interventions. Within the UK, National Health Service (NHS) gender services are provided by specialist gender identity clinics (GICs), which are currently primarily hosted by mental health trusts. Rates of referral to adult GICs in England have risen by 40% over the past 4 years,^1^ mirroring international trends.

## Background

‘Outcomes’ of interventions for gender dysphoria have been defined in various ways, including patient satisfaction, quality of life, body satisfaction, mental well-being and regret.^2–10^ Evidence is limited to cross-sectional, longitudinal and retrospective methods, often of low quality. Several systematic reviews of the evidence have concluded that quality of life^2^ and/or mental well-being^3–6^ improve following gender reassignment treatment. More recently, attention has turned to people who ‘detransition’ as an outcome. There is no universally accepted definition of ‘detransitioning’, though it broadly describes people returning to live in their original gender role, following a process of transition. This may or may not entail regret. Rates of detransitioning are unknown, with estimates ranging from less than 1%^11^ to 8%.^12^

A limitation of research to date has been to focus on those who complete transition, often being defined as having genital reconstructive surgery.^4^ Much less is known, either quantitatively or qualitatively, about those accessing gender services who do not access all of the interventions they seek. Although older studies observed that some people ‘dropped out’ of treatment, there is no contemporaneous data on this. There are limited data published by adult UK GICs,^13,14^ and to date there has been no requirement for services to collate or report on either access to treatment or outcomes. As such, it is unknown how many UK service users complete their transition as planned or have unmet needs, and the impact this may have on them. It is also unclear how many disengage from services, discontinue treatment or revert to their previous gender role.

As a response to the recent increased demand on services, UK gender services have been recommissioned to implement a new national service specification.^15^ The aim is to introduce uniformity of service provision and improve access, a central tenet of which is a streamlined assessment process and treatment pathway. It is envisaged that the majority of service users will require two appointments for an initial assessment to agree a diagnosis and devise a treatment plan based on the service users’ individual goals. The West of England GIC has been an early adopter of a model of care akin to the new service specification. As such, describing the patterns of service use and access to care by service users within this service has the potential to offer valuable insights in terms of national service development and resource allocation.

## Aims

A service evaluation was undertaken in the form of a retrospective case-notes audit of consecutively discharged patients, which aimed to: first, describe access to interventions compared with service users’ own goals, reasons for discharge, and re-referral patterns; second, to identify any factors associated with access to care in terms of background demographics and comorbidities; and, thirdly, to quantify the frequency of ‘detransitioning’. A lack of routine documentation on patient outcomes in terms of physical and psychological improvements from treatment meant that this could not be included.

## Method

### Service setting

The West of England GIC, located in Exeter, UK, is one of seven in England offering a national service for gender-diverse people aged 17 years and over; this includes those with both binary and non-binary gender identities. Throughout this paper, we use the terminology ‘natal male’ or ‘natal female’ to describe those whose sex at birth was assigned and registered as being, respectively, male or female but who identify as a different gender.

### Ethical approval

This work was undertaken as a service evaluation. It was confirmed with the Research and Development department within Devon Partnership Trust that ethics approval was not required and that the use of data was fully compliant with the Trust's privacy notice. The project was registered with the Quality Improvement department.

### Interventions and care pathway

NHS-funded interventions available are: hormonal (oestradiol, testosterone, GnRH analogue), masculinising chest surgery (partial mastectomy), gender reassignment surgery (GRS), facial hair removal, and speech and language therapy. Breast augmentation was funded until 2016. The standard care pathway during the study period comprised an initial assessment (usually by a psychotherapist) with or without further psychotherapy, then a diagnostic assessment by either a medical doctor (general practitioner [GP], sexual health physician or psychiatrist) or a psychologist, within which a treatment plan was agreed with the patient based on their own goals for transition. During treatment, participants were offered regular medical follow-up, and some chose to continue with psychotherapy. During August 2017, in anticipation of the new service specification, psychotherapists were replaced by ‘named professionals’ with a role more akin to that of a care coordinator.

### Data collection

We generated a list of all service users consecutively discharged by the GIC over a 12 month period (1 September 2017 to 31 August 2018). Service users who had not completed an assessment were excluded. We reviewed the electronic patient records for all service users. The GIC uses the same electronic system (Carenotes) as all secondary mental health services run by Devon Partnership Trust. These contain correspondence between the GIC and other services, GIC clinic letters, multidisciplinary team notes and clinical entries by GIC professionals. For Devon-based service users, any entries by other mental health teams are also contained in the records.

Data collection commenced in September 2018 and continued until December 2019, with all case notes undergoing a final review at that point in order to capture all subsequent information. The first 20 sets of notes were independently reviewed by two authors (R.E.H. and J.S.) in order to refine the data extraction tool; R.E.H. extracted the remaining data.

Referral age, natal sex, gender identity (including non-binary identities), and any previously diagnosed physical or mental health conditions or documented adverse childhood experiences (ACEs)^16^ were extracted from the electronic case records. ACEs were categorised as: physical abuse, sexual abuse, emotional abuse, bullying, parental separation, bereavement, witnessing domestic violence, parental mental health issues, parental substance misuse, living in care or living in poverty before age 18.

Information relating to interventions accessed, reasons for discharge and any unmet needs at discharge was extracted. Information on speech and language therapy was too sparsely documented to include. Psychotherapy was not collated as a distinct intervention, as provision changed during the period studied. Any relevant subsequent information or re-referral was also collected.

### Data analysis

Our primary outcome was the pattern of service use; we used descriptive statistics for rates of interventions sought and accessed, reasons for not accessing, reasons for discharge, and patterns of re-referral. We then grouped service users into either an ‘accessed care’ group or an ‘other outcome’ group. The ‘accessed care’ group comprised service users achieving all of their documented treatment goals (including where goals changed during treatment) and being discharged after completing treatment without rapid re-referral or evidence of detransitioning. The treatment pathway in the national service specification anticipates that care will be accessed in this way.^15^ All other service users were categorised as being in the ‘other outcomes’ group. Those transferring their care to another GIC were excluded from further analysis.

We then undertook a logistic regression, comparing the two groups in order to identify any predictors of different patterns of service use. As there were small amounts of missing data, most notably for social support, multiple imputation with 20 imputations was performed. This was followed by backward stepwise multivariable logistic regression, using a threshold *P*-value for retention of *P* < 0.05. The outcome variable was accessed care/other outcome, and all the remaining putative explanatory variables were entered. Stata 16 was used for the analyses.

Our secondary outcome was the frequency of detransitioning. In line with Richards,^11^ we defined this as those who had lived in an alternative gender role, reverting to their original role either during or after this care episode. Potential cases were flagged during data collection where there was any documentation of a change in gender role or gender identity, or discontinuation of treatment as reported by the service user, their GP or other third party. All authors discussed these anonymised cases to achieve consensus on how many met our case definition of detransitioning.

### Patient and public involvement

We discussed our findings with a future service user of the West of England GIC. She contributed ideas to the significance and relevance of the findings to service users in the capacity of a lived experience consultant. She also commented on an early draft of the paper.

## Results

### Participants

There were 182 service users discharged between 1 September 2017 and 31 August 2018. One set of notes was excluded as it was an information-only request, and six others were excluded as they had not completed their assessment with the service (three disengaged, two had significant mental health concerns and one sought private care elsewhere). The remaining 175 service users included 67 (38%) natal females and 108 (62%) natal males. The median age was 25 years overall; 36 years for natal males and 20 years for natal females. They had been referred between 2010 and 2017.

At diagnostic assessment, most expressed a binary trans identity: natal male to female (*n* = 101, 57.7%) or natal female to male (*n* = 59, 33.7%). A smaller number were natal female to a non-binary identity (*n* = 8, 4.6%) or natal male to a non-binary identity (*n* = 6, 3.4%). One service user, natal male, had previously transitioned to female (at a different GIC) and was referred to medically ‘detransition’.

Table 1 shows rates of physical and mental health issues, which had been diagnosed elsewhere prior to assessment. One or more physical health issue was documented for 79 service users (45%), including obesity for 26 (14.9%). At least one previously diagnosed mental health condition was documented for 126/174 service users (72.4%), including 110/174 (63.2%) with anxiety and/or depression, 12/174 (6.9%) with personality disorder and 4/174 (2.3%) with a suspected personality disorder, and 7/174 (4%) with an eating disorder. Neurodevelopmental disorders (attention-deficit hyperactivity disorder, autism spectrum conditions, dyslexia or dyspraxia) were diagnosed for 22/173 (12.7%) service users with adequate documentation; these diagnoses were primarily among those under 25 years old (18/86, 20.9%).

**Table 1:** Service user background characteristics

	All service users (*N* = 175) No. cases/total (excluding incomplete notes)	Natal females		Natal males	
		<25 y (*N* = 48)	>25 y (*N* = 19)	<25 y (*N* = 39)	>25 y (*N* = 69)
Previous mental health diagnoses	126/174 (72.4%)	37/48 (77.1%)	15/19 (78.9%)	32/39 (82.1%)	42/68 (61.8%)
Any neurodevelopmental disorder	22/173 (12.7%)	11/47 (23.4%)	1/19 (5.3%)	7/39 (17.9%)	2/68 (2.9%)
Any previous mental health service use (private or NHS)	106/174 (60.9%)	36/48 (75.0%)	12/19 (63.2%)	26/39 (66.7%)	31/68 (45.6%)
Previous NHS secondary mental health service or CAMHS use	67/174 (38.5%)	29/48 (60.4%)	9/19 (47.4%)	17/39 (43.6%)	12/68 (17.6%)
History of drug or alcohol misuse	38/173 (22.0%)	11/47 (23.4%)	6/19 (31.6%)	8/39 (20.5%)	13/68 (19.1%)
History of self-harming	74/173 (42.8%)	36/50 (72.0%)	10/19 (52.6%)	19/39 (48.7%)	10/68 (14.7%)
History of suicide attempt(s)	44/173 (25.4%)	12/48 (25%)	10/19 (52.6%)	12/39 (30.8%)	11/68 (16.2%)
1+ adverse childhood experiences	128/169 (75.7%)	42/47 (89.4%)	15/18 (83.3%)	27/39 (69.2%)	44/65 (67.7%)
3+ adverse childhood experiences	37/169 (21.9%)	17/47 (36.2%)	4/18 (22.2%)	7/39 (17.9%)	9/65 (13.8%)
1+ coexisting physical health condition(s)	79/175 (45.1%)	20/48 (41.7%)	14/19 (73.7%)	10/39 (25.6%)	35/69 (50.7%)
Documented social support	121/153 (79.1%)	37/43 (86.0%)	14/16 (87.5%)	23/35 (65.7%)	47/60 (78.3%)

CAMHS, child and adolescent mental health services; NHS, National Health Service.

As shown in Table 2, 73 service users (42.9%) had concerns about their mental health documented during treatment, with just under half of these being significant enough to meet the threshold for accessing secondary NHS mental health services. There were three completed suicides in people accessing treatment.

**Table 2:** Mental health (MH) during treatment

	All service users (*N* = 175) No. cases/total (excluding incomplete notes)	Natal females		Natal males	
		<25 years old (*N* = 48)	>25 years old (*N* = 19)	<25 years old (*N* = 39)	>25 years old (*N* = 69)
Documented MH concerns during treatment	73/170 (42.9%)	26/46 (56.5%)	14/15 (93.3%)	2/39 (5.1%)	16/66 (24.2%)
Documented contact with MH services during treatment	45/173 (26.0%)	17/46 (37.0%)	5/19 (26.3%)	12/39 (30.8%)	11/69 (15.9%)
MH contact recommended but not received during treatment	15/173 (8.7%)	7/46 (15.2%)	3/19 (15.8%)	3/39 (7.7%)	2/69 (2.9%)
Contact with secondary MH services during treatment	31/173 (17.9%)	12/46 (26.1%)	3/19 (15.8%)	9/39 (23.1%)	7/69 (10%)
Documented self-harm during treatment	25/172 (14.5%)	14/48 (29.2%)	2/18 (11.1%)	8/39 (20.5%)	1/68 (1.5%)
Documented suicide attempts during treatment	14/173 (8.1%)	5/48 (10.4%)	0/18 (0.0%)	6/39 (15.4%)	3/68 (4.4%)
Drug or alcohol misuse during treatment	18/173 (10.4%)	7/47 (14.9%)	0/19 (0.0%)	5/39 (12.8%)	6/68 (8.8%)
Completed suicide during treatment	3/175 (1.7%)	0/48 (0.0%)	0/19 (0.0%)	3/39 (7.7%)	0/69 (0.0%)

### Main outcomes

**Fig. 1:**
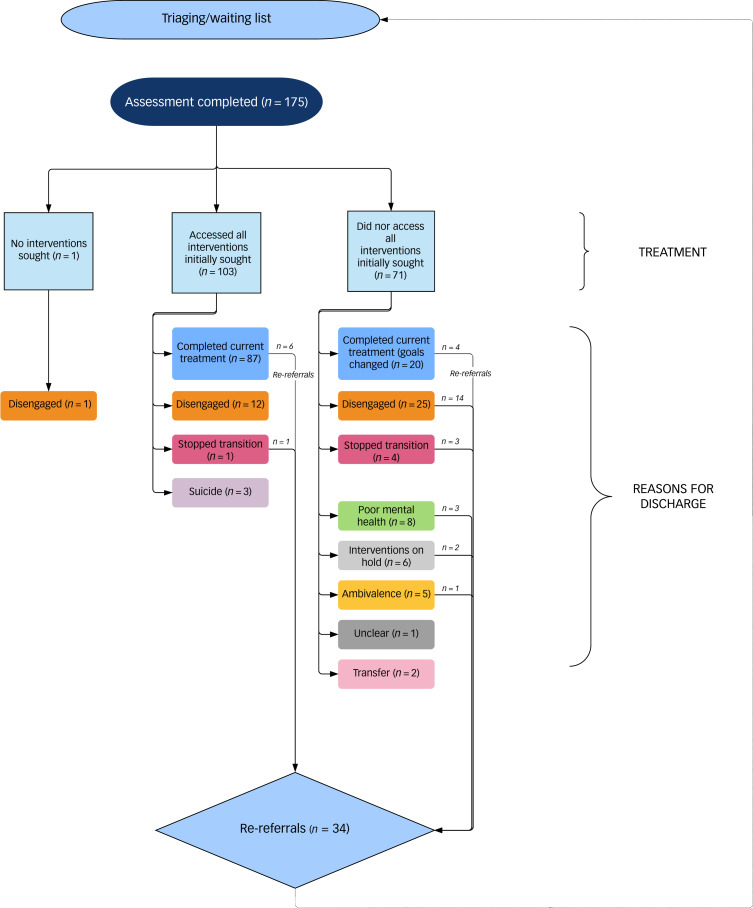
Access and patterns of service use.

Figure 1 summarises whether all interventions were accessed, reasons for discharge and numbers to be re-referred.

### Interventions accessed

One participant was not seeking any interventions. We found that 103 out of 174 participants (58%) accessed all of the interventions they initially sought. Hormone treatments were the most commonly accessed where originally sought, and masculinising GRS was the least commonly accessed. Table 3 summarises the interventions that were sought and accessed by service users.

**Table 3:** Interventions sought and accessed

Intervention	No. accessing/no. seeking	Reasons for not accessing
Oestradiol (starting or continuing under GIC management)	94/101 (93.1%)	No transition (*n* = 1)
		GP refused to prescribe (*n* = 1)
		Other provider (*n* = 1)
		Ambivalent (*n* = 1)
		Disengaged (*n* = 2)
		Unclear (*n* = 1)
Testosterone (starting or continuing under GIC management)	62/65 (95.4%)	Fertility prioritised (1)
		Disengaged (1)
		Other provider (1)
Masculinising chest surgery	57/65 (87.7%)	Disengaged (4)
		Physical health (1)
		Mental health (1)
		Ambivalent (1)
		De- and retransitioned (1)
Breast augmentation	3/8 (37.5%)	Unclear (1)
		Funding cut (4)
Facial hair removal	81/94 (86.2%)	Patient choice (3)
		No providers (2)
		No transition (3)
		Unclear (5)
Feminising genital reconstructive surgery	43/76 (56.6%)	See ‘Gender reassignment surgery’ section below
Masculinising genital reconstructive surgery	8/31 (25.8%)	See ‘Gender reassignment surgery’ section below
All interventions	103/174 (59.2%)	

GIC, gender identity clinic; GP, general practitioner.

### Gender reassignment surgery

Feminising GRS was completed by 43 out of 76 (56.5%) service users initially seeking it, with three of these people obtaining it privately. The documented reasons for 33 participants not achieving GRS were: disengagement (*n* = 8), service user choice (*n* = 8), poor mental health (*n* = 3), physical health condition (*n* = 3), ambivalence (*n* = 3), stopped transition (*n* = 4), did not transition (*n* = 2) or unclear (*n* = 2).

Masculinising GRS was completed by eight out of the 31 (25.8%) service users initially seeking it. Reasons for 23 people not achieving masculinising genital surgery were: administrative error (*n* = 1), ambivalence (*n* = 1), disengagement (*n* = 6), poor mental health (*n* = 4), did not transition (*n* = 1), service user choice (*n* = 5), physical health condition (*n* = 4) or social reasons (*n* = 1). There were 16 service users who were unsure at diagnosis; two subsequently accessed surgery.

### Reasons for discharge

There were a number of reasons for service users being discharged from the service. They can be summarised as: (a) those who ‘completed current treatment’, including those who did not access all interventions initially sought as they changed their goals or accepted limitations (*n* = 107, 61.1%); (b) those who disengaged and were discharged (*n* = 38, 21.7%); (c) those whose mental health was too poor to progress (*n* = 8, 4.5%); (d) those who stopped transition (*n* = 5, 2.8%); (e) those who continued to be ambivalent about the interventions sought and unable to move forward with transition (*n* = 5, 2.8%); (f) those who had clear goals for further interventions but had to ‘pause’ as they were unable to pursue them currently for social or health reasons (*n* = 6, 3.4%); (g) completed suicides (*n* = 3, 1.7%); (h) transfer to other GICs (*n* = 2, 1.1%); and (i) unclear (*n* = 1, 0.6%).

#### Re-referrals

Following discharge, 34 service users were re-referred (19.4%) to the GIC within the 16 month period of data collection. Ten of the re-referrals had been discharged after completing current treatment goals and were re-referred due to: seeking GRS (6/10), poor mental health (1/10), detransitioning (1/10), hormone compliance issues (1/10) and treatment dissatisfaction (1/10). The majority of those re-referred (24/34, 70.6%) had not completed current treatment.

Seven of the 34 re-referrals were discharged for a second time during the period of data collection due to: service user requesting discharge (*n* = 4), non-attendance (*n* = 2) or unstable mental health (*n* = 1).

### Analysis of service outcomes

**Fig. 2:**
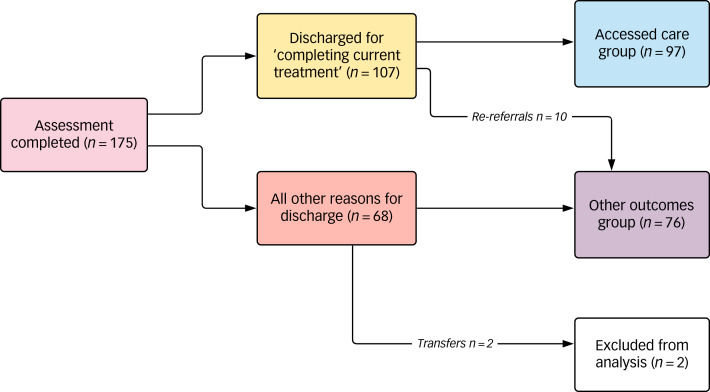
Groups for logistic regression.

The logistic regression included 173 patients. There were 97 (56.1%) service users in the ‘accessed care’ group, comprising all those discharged for ‘completing current treatment’ minus the ten who were subsequently re-referred. There were 76 (43.9%) in the ‘other outcomes’ group, which comprised all other reasons for discharge except the two transfers, who were excluded. Fig. 2 summarises this.

According to the univariate analyses, several variables were associated with an accessed care or other outcome. However, the multivariate analyses found that only four variables (neurodevelopmental disorders, substance misuse during treatment, mental health concerns during treatment and number of ACEs) were independently associated with outcome (see Table 4). A dose-response relationship to the number of ACEs was observed.

**Table 4:** Logistic regression

	Univariate analysis			Multivariate analysis		
	Odds ratio	95% CI	*P*-value	Odds ratio	95% CI	*P*-value
History of drug or alcohol misuse	1.8	0.9 to 3.7	0.11			
Secondary mental health service use during treatment	3.3	1.5 to 7.6	0.004			
Previous mental health diagnosis	2.1	1.0 to 4.2	0.044			
Age at referral^a^	0.97	0.95 to 0.99	0.008			
Social support	0.59	0.27 to 1.3	0.20			
Previous use of CAMHS or secondary mental health services	3.5	1.8 to 6.6	<0.001			
Living full-time in social role	1.2	0.60 to 2.5	0.56			
History of self-harm	2.0	1.1 to 3.7	0.03			
Natal sex	0.43	0.23 to 0.80	0.008			
Neurodiversity	7.3	2.3 to 23	0.001	5.7	1.7 to 19	0.005
Drug or alcohol misuse during treatment	7.7	2.2 to 28	0.002	4.3	1.1 to 17.6	0.040
Mental health concerns during treatment	3.4	1.8 to 6.5	<0.001	2.2	1.1 to 4.4	0.033
Number of ACEs^a^	1.6	1.3 to 2.2	<0.001	1.5	1.1 to 1.9	0.011

ACE, adverse childhood experience; CAMHS, child and adolescent mental health services.

a. As the explanatory variable is a continuous variable, this odds ratio is per unit change (year; number of ACEs).

### Secondary outcome: detransitioning

Twenty-one sets of notes out of the 175 were flagged as potential cases of detransitioning for consensus discussion. Three cases were excluded following the consensus discussion; one postponed both medical and social transition until they had more social support, and two discontinued hormones but did not revert to their original gender role.

Twelve cases (12/175, 6.9%) were agreed by all authors to meet the case definition for detransitioning. Regret was specifically documented in two cases. Eight were natal males (seven male to female, one male to non-binary); all had accessed oestradiol and one had accessed GRS. Four were natal females (three female to male, one female to non-binary); all had accessed testosterone and chest surgery during this episode of care, none had accessed GRS. Nine of the twelve had evidence of discontinuing hormones, two had no information documented about hormones and one continued with hormones. Four of these 12 were re-referred into the service during the period of data collection since de-transitioning.

Six cases did not strictly meet the criteria for detransitioning but showed some overlap of experience. One of these six has been re-referred. Four natal males (three male to female, one male to non-binary) had made only partial role transitions so did not meet the case definition; they inconsistently used hormonal interventions and expressed uncertainty about their gender and/or transitioning. Two natal females (one female to male, one female to non-binary) expressed gender identity confusion, one used testosterone inconsistently and both cancelled chest surgery; neither, however, clearly reverted back to their original gender role and therefore did not meet the case definition.

## Discussion

### Principal findings

Overall, 59% of service users accessed all of the interventions they initially sought. Most accessed hormones where sought (94%), though fewer accessed gender reassignment surgery; 57% accessed feminising and 26% masculinising GRS where originally sought. We observed that some service users changed their treatment goals during their time within the service; however, the main reasons for GRS not being accessed were disengagement, poor mental or physical health (including obesity) and social constraints. More than one in five (21.7%) service users disengaged from the service and were discharged for non-attendance. In addition, 19.4% were re-referred in a relatively short space of time and re-joined the waiting list.

Just over half of our service users (56.1%) completed the treatment pathway in line with the service model. When we compared the ‘accessed care’ group with all ‘other outcomes’, we found several factors associated with these divergent service outcomes. The presence of a neurodevelopmental disorder, adverse childhood experiences (as a dose response), substance misuse during treatment and mental health issues during treatment were all independently associated with ‘other outcomes’.

Twelve service users (6.9%) met our case definition of detransitioning. A further six (3.4%) service users had some overlap of experience though they did not strictly meet the case definition.

### Strengths and limitations

The population accessing this service, as a national clinic, is likely to be representative of the population accessing GICs across the UK. This is the first retrospective review of consecutively discharged patients from a UK GIC and the first service evaluation to describe the patterns of service use. Data on numbers of service users completing their transition in line with their self-defined goals were previously unknown. The additional pressure on waiting lists from re-referrals has not previously been described and needs addressing.

This evaluation was limited by its reliance on clinical notes, which had some missing information. As the GIC does not itself diagnose concurrent mental or physical health issues, we relied on the documentation of diagnoses made elsewhere, meaning our background characteristics data may be underestimates. The logistic regression relied on routinely collected information; there may be important variables which we could not examine.

We defined our primary outcome according to the service model. In comparing groups, we implied that the ‘accessed care’ outcome was the more favourable. However, whether this is the case from a service user perspective remains unknown. Patient satisfaction levels might be similar irrespective of which group service users fall into; these data are not routinely collected. A further limitation was not being able to quantify the outcomes for service users in terms of mental and physical health improvements compared with baseline owing to a lack of data. At present there is no standardised approach to measure and record such outcomes.

As data collection occurred for only 16 months after the most recent discharge, we may have underestimated the frequency of detransitioning. There is some evidence that people detransition on average 4^17^ or 8 years^18^ after completion of transition, with regret expressed after 10 years.^10^ Furthermore, as there is no automatic mechanism to inform GICs of service users who subsequently detransition, other instances may have been missed. We gleaned only a limited understanding of those who detransitioned, owing to our reliance on notes. Regret was specifically documented in two cases but may or may not have been experienced by others too. Conversely, the process of transitioning and subsequently detransitioning may, in its own right, have been a positive experience for some

### Comparison with other studies

The demographics of our cohort were similar to those described elsewhere, comprising younger natal females and relatively older natal males accessing services, probably reflecting shifts in sex ratios seen in adolescent clinics.^19,20^ The high levels of previous mental health problems also reflect what has been described elsewhere, with rates above those of the general population.^6,21,22^ We do not know whether the three completed suicides we reported were comparable with rates in other UK clinics; however, higher rates of suicide among trans people are well recognised, with the increased risk observed to persist at all stages of transition.^23^

There is a dearth of prospective studies and no controlled prospective studies.^4^ Equally longitudinal studies suffer from loss to follow up. A problem arising from this is that little is known about all possible outcomes of people accessing gender services and limited data with which to compare the pathways we have described. An older Dutch study reported 15% of those starting cross-sex hormones subsequently ‘dropped out’ and stopped hormones.^24^ Similar to our findings for not completing the treatment pathway, a risk factor for ‘dropping out’ was poor psychological functioning. Studies on dissatisfaction with treatment have also highlighted the association with poor baseline psychological functioning.^25^

There are limited comparable data on rates of accessing interventions. A Dutch study^10^ reported that 68.9% of adults started cross-sex hormones in the 5 years following diagnosis, lower than our finding of 94% accessing hormones. However, the same study found a higher rate of progression to GRS (77.7% compared with our 47.7%). An older UK study reported an even higher rate of progression to GRS of 94%.^13^ Our lower rate of accessing GRS might reflect changes in the demographics of service users across time; the service users in the older UK study were predominantly male to female. Another possible explanation for differences in accessing interventions is differing rates of diagnosis of gender dysphoria. Khoosal et al^13^ reported that 77% met the diagnostic criteria; we do not have a comparative figure for the West of England GIC, but potentially a higher proportion of those assessed during our study period could have been diagnosed with gender dysphoria. An alternative explanation for our low GRS rates might be inaccurately elicited treatment goals. A previous study highlighted the tendency of patients to say they were seeking GRS as they assumed that GICs expected to hear this.^26^ It is also possible that the association we observed between mental health issues and substance misuse during transition and not accessing care is mediated by clinician bias in reluctance to refer these service users for surgery; this warrants further exploration.

Notwithstanding the possibility that the rate of detransitioning we found (6.9%) is an underestimate, it is notably higher than the only other published figure from a UK clinic of 0.33%^11^ despite using the same case definition. This likely reflects methodological differences insofar as we looked at patients discharged by the GIC and had access to subsequent information over a 16 month period rather than looking only at service users in treatment. A US survey-based study of people identifying as transgender described patterns of detransitioning and then attempts to retransition akin to our observations.^12^

### Implications and conclusions

Different GICs in the UK have historically used different service models. The new service specification^15^ will be adopted by all GICs and is similar to the model already used by the West of England GIC. Our findings suggest that caution might be warranted in the uniform introduction of the new assessment and treatment pathways, as many did not access the care they sought. The heterogeneity of the population accessing gender services needs adequate recognition and accommodation, with more flexible and individualised care pathways and in conjunction with other services. The traditional model of care in adult GICs is based on experience with older transwomen, not younger transmen or non-binary service users. There is a need to better understand the specific needs of this new younger generation of service users and shape services accordingly, while simultaneously not disadvantaging those who may benefit from a streamlined assessment approach.

Given our finding of an association between neurodevelopmental disorders and ACEs and not accessing care, we would advocate careful attention to these factors during assessment. Agreeing realistic goals and considering any necessary adaptations to the treatment pathway is vital if service users’ needs are to be met. Consent to irreversible treatments must entail a discussion about the real possibility of not completing transition as envisaged, in order that expectations are managed. It is not currently known what it means for service users to access hormones but not surgery where desired. Furthermore, it is necessary to optimise support for those with coexisting substance misuse or mental health concerns during treatment.

Our service evaluation has revealed a number of issues demanding research. First, what constitutes a ‘good’ outcome from a gender clinic and how this might be measured. Second, why service users disengage or rapidly seek re-referral post discharge, especially given the long waiting times at GICs (over 6 years to diagnostic assessment at West of England GIC prior to COVID-19; personal communication, Maria Morris, 2020). Finally, understanding those who stop or reverse their transition. It may be more helpful to think about these people less in terms of whether they are ‘detransitioners’ or not but as representing a spectrum for whom transition is not a finite, linear experience but entails change along the way. Qualitative research is needed to understand these experiences. Meaningful service user involvement by trans people in all such research and ongoing service development is paramount.^27^

As services are reconfigured, there is a unique opportunity for more coordinated and standardised data collection and reporting to be implemented across services. This would allow for benchmarking both nationally and internationally. At present, the lack of follow-up of service users following their discharge from a GIC means that services are not necessarily aware of longer-term outcomes (such as detransitioning or suicide) and so lack assurance that they are providing high-quality services. We recognise the current barriers to routine long-term follow-up; however, without this longer-term outcomes will remain poorly understood and the health needs of trans people may not be met.

## Data Availability

The data are not publicly available due to this being a service evaluation and their containing information that could compromise the privacy of service users.

## Supplementary material

For supplementary material accompanying this paper visit http://dx.doi.org/10.1192/bjo.2021.1022. click here to view supplementary material
